# Capture‐enhanced neutron irradiation to treat Alzheimer's disease: Design of a small animal set‐up for future in‐vivo experiments

**DOI:** 10.1002/mp.18062

**Published:** 2025-09-01

**Authors:** Valeria Pascali, Davide Tosoni, Saverio Altieri, Nicoletta Protti

**Affiliations:** ^1^ Department of Physics University of Pavia Pavia Italy; ^2^ National Institute of Nuclear Physics INFN Pavia Unit Pavia Italy

**Keywords:** Alzheimer's disease, computational dosimetry, irradiation set‐up, neutron activation, neutron capture reaction

## Abstract

**Background:**

Alzheimer's disease (AD) is characterized by the accumulation of β‐Amyloid and τ proteins in the brain that causes dementia. To date, there is no cure capable of eradicating AD, so it is necessary to study a performing therapy. The NECTAR project aims to investigate an extension of the conventional Boron Neutron Capture Therapy principles as a possible treatment for AD at different scales (protein, cells, animal).

**Purpose:**

The present study focuses on a macroscopic scale and wants to propose an irradiation set‐up for mice in the thermal column (TC) of the Triga Mark II reactor of Pavia University, in view of the forthcoming in vivo irradiation of healthy and transgenic AD mouse models.

**Methods:**

Monte Carlo simulations were carried out with the MCNP6 code to test different irradiation positions and study the least toxic treatment possible by modeling neutron shielding to preserve healthy tissue. A shielding prototype was built and tested by means of neutron activation measurements. A geometrical mouse model was developed with the aim of computing the dose‐rates induced in each radiosensitive organ and thus to estimate possible irradiation times for future in vivo experiments.

**Results:**

The computational study showed that the safest irradiation condition involves placing the shielding 20 cm from the TC entrance and that the best performing shielding material is 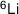 enriched lithium carbonate. Furthermore, taking into account the tolerance doses of each organ, the maximum animal irradiation time in an AD context is 45 min. The proposed set‐up could also be used for preclinical studies on brain tumors; in this context, the maximum estimated irradiation time is 11 min.

**Conclusion:**

The proposed work is pivotal in the study of a possible treatment for AD in a neutron irradiation context, paving the way for the next phase of the NECTAR project involving in vivo irradiation of AD mouse models and thus making it possible to assess its efficacy and its possible future extension to the human brain.

## 1 INTRODUCTION

Alzheimer's disease (AD) is the most common form of dementia, a neurodegenerative disorder characterized by a progressive accumulation of the β‐Amyloid (Aβ) protein in the brain's extracellular matrix and the τ protein within neurons, which damages brain cells.^1^

There are currently around 55 million people suffering from AD, and although it has been known for many years, there are still no drugs able to eradicate it, only a few treatments that can alleviate its symptoms.^2^ Over time, several researches have been conducted, most of them aimed at developing β‐Amyloid and/or τ protein inhibitors to help reduce protein aggregates.^3^, ^4^ In June 2021 and January 2023 two monoclonal antibodies, aducanumab and Lequembi,^5^, ^6^ were approved by the US Food and Drug Administration (FDA). Both drugs are claimed to be capable of progressively slowing down the development of Alzheimer's disease by acting on the reduction of Aβ aggregates. Nonetheless, their use is not without controversy: in fact, aducanumab has not been approved by the European Medicine Agency (EMA) due to mixed results collected from two major clinical trials, particularly in terms of side effects.^7^, ^8^

In recent years, evidences have been accumulated about the beneficial effects of conventional radiotherapy on Alzheimer's disease. In particular, in vitro^9^ and in vivo studies in AD mouse models^10^, ^11^ have shown the efficacy of x‐ray radiation in reducing Aβ aggregates accompanied, in the case of animal irradiation, by the absence of side effects. Cognitive and behavioral improvements have also been observed in pilot studies carried out on patients with early‐stage and advanced AD^12^, ^13^, ^14^, ^15^ following a series of CT scans.

Considering the above, a beneficial role of radiation in slowing down and/or blocking disease progression through the activation of the glia cells, is clearly evident.

The NECTAR (NEutron Capture enhanced Treatment of neurotoxic Amyloid aggRegates) project,^16^ funded by European Community H2020 framework aims to further investigate the effect of ionizing radiations on AD. In particular, it explores the effectiveness of a treatment based on a Capture‐Enhanced Neutron Irradiation (CENI) for Alzheimer's disease and the innovative idea is to exploit the basic principles of neutron capture therapy^17^ (NCT) to investigate the highly localized action of the 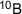 and 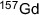 neutron capture reactions in the depolymerization of β‐amyloid aggregates. The high selectivity of these reactions is made possible by the selective binding of boron carriers to β‐amyloid aggregates, which enables a chemically targeted delivery of the neutron capture agents and, consequently, a spatially confined emission of high‐LET charged particles whose ranges are well matched to the typical dimensions of Aβ aggregates (from a few nanometers up to millimeters). This action is intended to be combined with that of the photons produced by the same reactions which, acting over long distances, could activate microglia cells (immune compartment of the brain) and thus promoting the phagocytosis of the Aβ aggregates, as already observed in the application of conventional radiotherapy in AD models.

NECTAR project is essentially a pre‐clinical research focused on the proof of concept in transgenic AD animals of the feasibility, safety and effectiveness of CENI as an innovative treatment of AD. In this respect, at the Physics Department of the University of Pavia, a study is being conducted in order to develop an irradiation set‐up for small animals to be used inside the thermal column of the TRIGA Mark II research nuclear reactor, housed at the Applied Nuclear Energy Laboratory (L.E.N.A.) of the University of Pavia.

In this work, different irradiation positions and shielding materials have been evaluated and are discussed; in addition, a geometrical mouse model has been developed using the Monte Carlo code MCNP6 to estimate the doses deposited in the animal's organs during a CENI treatment. A preliminary validation of the developed irradiation set‐up has been carried out through experimental neutron activation measurements.

## 2 MATERIALS AND METHODS

**FIGURE 1:**
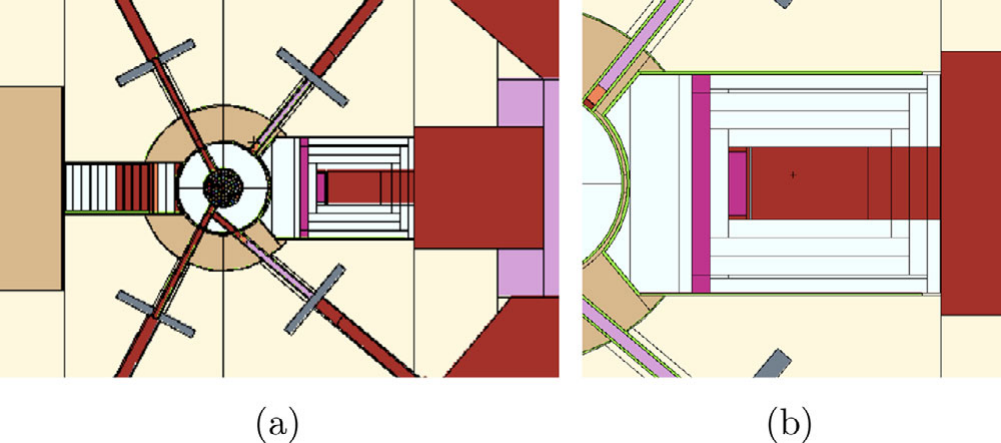
(a) Top cross‐sectional view of the Triga Mark II nuclear research reactor obtained with MCNP6; the core is visible at the center, with four irradiation channels extending outward. On the right, the thermal column is shown in red. (b) Zoomed‐in top view of the thermal column of the Triga Mark II reactor (100 x 20 x 40 ${\mathrm{cm}}^{3}$), obtained with MCNP6.

The TRIGA Mark II research nuclear reactor of the University of Pavia features a modified thermal column^18^ that houses a 100 x 20 x 40 ${\mathrm{cm}}^{3}$ cavity with an uncollimated field of thermal neutrons slightly contaminated by epithermal and fast neutrons as well as photons^19^, ^20^, ^21^ (Figure 1 ab). For several years, the cavity has been used to conduct BNCT experiments including in‐vivo irradiations of small animals (rats, mice).^19^, ^22^ The BNCT small animal experiments carried out so far always exploited the innermost part of the irradiation cavity, where an in air thermal neutron flux of the order of 10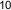 n/${\mathrm{cm}}^{2}/s$ is present. The contamination by epithermal and fast neutrons are, respectively, 2 and 3 order of magnitude lower.^23^ The in air total flux reduces of one order of magnitude inside the animal body under direct neutron irradiation if proper 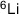
‐enriched lithium carbonate shields are used. Indeed the longest experience on in vivo experiments at Pavia reactor deals with the effectiveness of BNCT in treating different types of lung cancers thus the sizes of the available shields work well in case of a target localized more or less in the middle of animal body. As consequence, these shields represent a limitation more than a sparing tool due to the target in the brain of the NECTAR project. In addition to what said, the innermost position of the irradiation chamber is affected by a quite huge dose rate due to background photons (mainly coming from the neutron activation of the materials composing the thermal column, i.e., graphite and a couple of bismuth blocks to attenuate the photon contamination coming from the reactor core^23^) thus the possibility of reducing this non‐selective contribution with the aim of minimizing the whole‐body dose must be evaluated. Considering all this and in perspective of the new experimental campaigns with animals foreseen by the NECTAR project, a revision of the described irradiation set‐up has been carried out with the main goals of (a) reducing the undue dose to healthy tissues and (b) minimizing irradiation times. Considering the exploitation of the L.E.N.A. thermal column for the NECTAR project as well as for BNCT anti‐cancer purposes, the present study evaluates the macroscopic dosimetry in the animal body and organs both with the aim of treating the animal brain affected by AD and bearing cerebral tumors.

This study was conducted through Monte Carlo simulations carried out with the MCNP6 particles transport code^24^ where the model of the TRIGA Mark II reactor of the University of Pavia was implemented several years ago.

### 2.1 Design of a neutron shield prototype and flux estimation at the irradiation positions of the Pavia reactor thermal column

**FIGURE 2:**
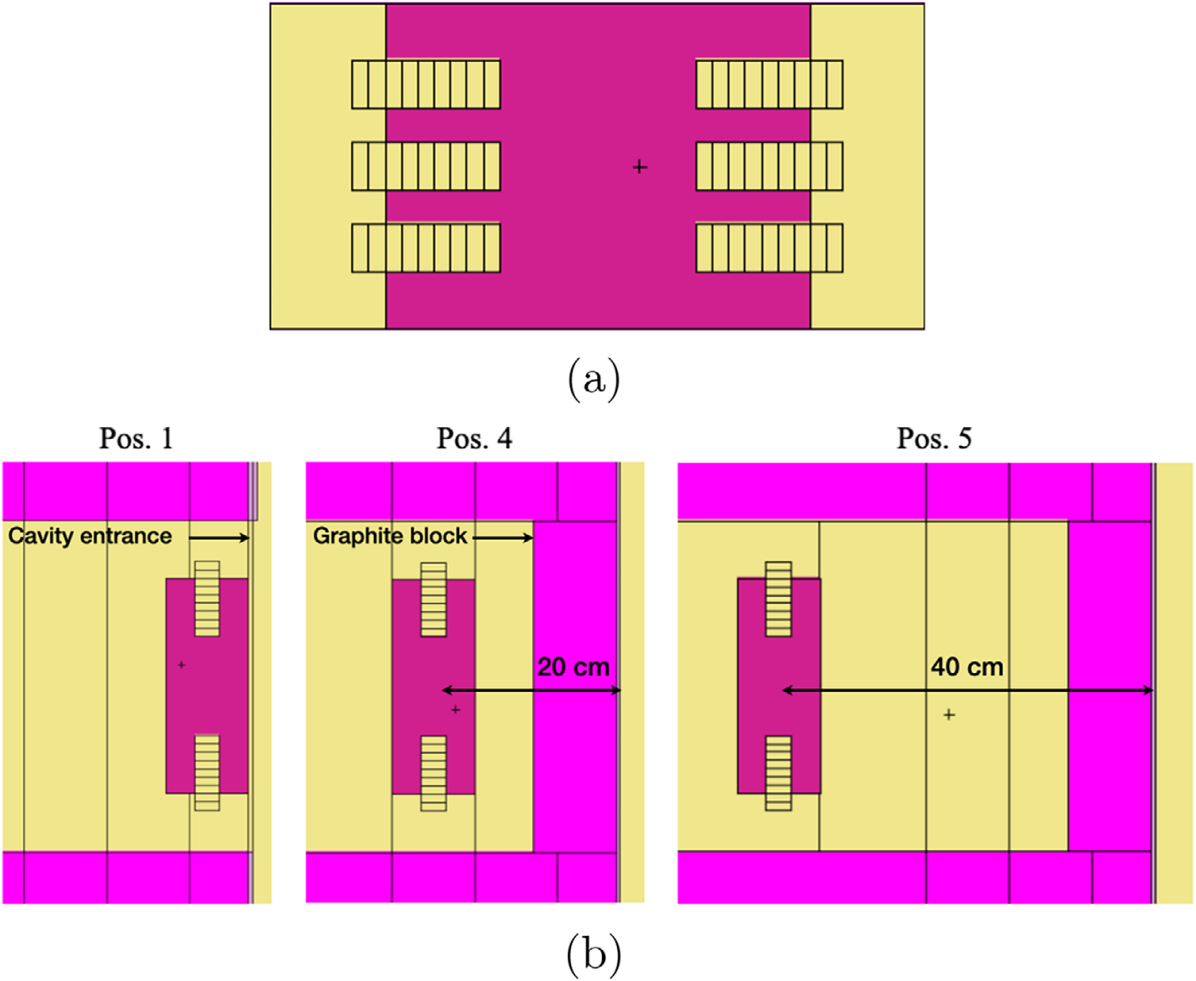
(a) Shield set‐up (frontal view) designed in MCNP6 geometry with six lateral holes to allocate six mice simultaneously in future experiments. To perform a first fluxes characterization, small air cylinders were used to model the different mouse body regions for each of the six holes; specifically: the two protruding outwards represent the head, the central four the main body and the last two the caudal region; (b) shield positioned at irradiation positions 1 (with the outer edge coinciding with the thermal column entrance), 4 (20 cm from the cavity entrance) and 5 (40 cm from the entrance). A 40×20×10 ${\mathrm{cm}}^{3}$ graphite block was added in pos. 4 and pos. 5 configurations.

The search for a new irradiation set‐up made it necessary to model a new neutron shield in the geometry of the MCNP code, version 6.1, to preserve healthy animal tissues. After designing several prototypes with different geometries, the most effective configuration was a 13×20×10 ${\mathrm{cm}}^{3}$ parallelepiped, in which six holes were created on the two lateral faces to allow the simultaneous irradiation of six mice in future experiments (Figure 2a). Specifically, three vertical cylindrical holes with a diameter of 3 cm (corresponding to the average size of a mouse) were drilled on each lateral face of the shield.

Three different shielding materials were tested: lithium polyethylene, lithium carbonate, and lithium fluoride, all enriched in 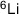 since it has a high thermal neutron capture cross section (941 barn at 0.025 eV) leading to the production of an alpha particle and tritium with a *Q*‐value of 4.78 MeV. The composition of the materials considered is described in Table 1.

**TABLE 1:** Atomic composition and density of shielding materials.

	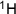	C	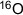	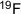	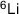	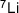	Density
Material	Weight (%)						(g/${\mathrm{cm}}^{3}$)
Lithium polyethylene	8.6	84.12	—	—	6.916	0.364	1.06
Lithium carbonate	—	16.26	64.96	—	17.841	0.939	1.72
Lithium fluoride	—	—	—	73.24	25.58	1.18	2.635

In order to evaluate the neutron and gamma fluxes, an elementary mouse model was considered, developed using nine different cylinders with a diameter of 3 cm, two of which represent the head, five the central body and the last two the caudal region (Figure 2a). Evaluations were carried out in air to investigate whether the actual neutron flux at the brain meets the minimum intensity requirement for a standard NCT treatment of 10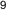 neutrons/${\mathrm{cm}}^{2}$/s.^17^ A further evaluation was made considering tissue to assess neutron absorption due to capture reactions on hydrogen and nitrogen.

In the Monte Carlo simulations conducted, three different irradiation positions were evaluated: (i) shield with the outer edge coinciding with the entrance of the thermal column (pos. 1); shield placed approximately (ii) 20 cm (pos. 4), and (iii) 40 cm (pos. 5) from the cavity entrance (Figure 2b). To reduce the 478 keV gamma contribution coming from the neutron capture reactions occurring at the boral screen covering the cavity entrance, a 40 x 20 x 10 ${\mathrm{cm}}^{3}$ graphite block was placed between the shield and the screen (Figure 2b, pos. 4 and pos. 5).

### 2.2 Computational dosimetry and neutron activation using a geometrical mouse model

A mouse model was implemented in the MNCP6 geometry to develop a preliminary treatment plan in a Capture‐Enhanced Neutron Irradiation context for future in vivo irradiation within the NECTAR project. This model was used to calculate the absorbed dose‐rates delivered to the animal's organs due to the interaction of the n+γ field with the tissues and thus to identify the irradiation parameters to deliver a safe and tolerable irradiation to the animal.

**FIGURE 3:**
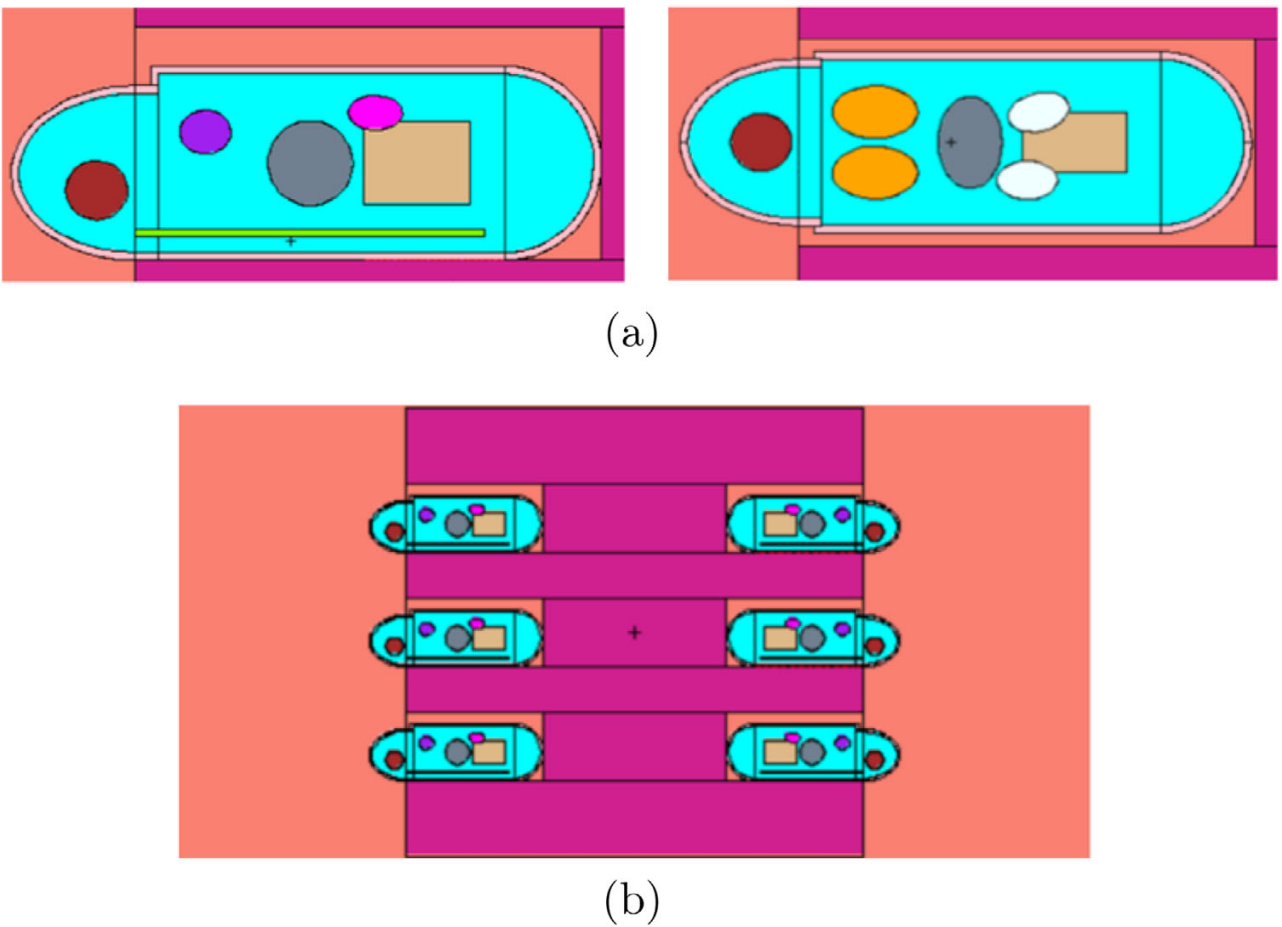
(a) Lateral and top view of the mouse model designed in the MCNP geometry (*yz* plane); (b) Frontal view of the designed neutron shield with the six mice models.

The body of the mouse (Figure 3a) was modeled as a cylinder with a diameter of 2.5 cm and a length of 6 cm. A semi‐ellipsoid and a hemisphere were used to simulate the head and the end of the body (sacral area), respectively. Most of the mouse radiosensitive organs were added: brain, lungs, hearth, spinal cord, liver, stomach, kidneys, and intestine. The rest of the body was assumed to consist of soft tissue.

Since animal tissues are not very different from human tissues, the weight percentages reported by ICRU (International Commission on Radiation Units and Measurements), referred to human tissues and accepted by the particle therapy community, were considered.^25^, ^26^ Table 2 shows the compositions and densities of each tissue and the mass of each organ.

**TABLE 2:** Mouse tissues composition and density.

	Density ($g/cm^{3}$)	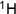 (%)	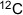 (%)	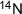 (%)	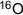 (%)	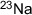 (%)	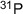 (%)	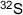 (%)	Cl (%)	K (%)
Brain	1.04	10.7	14.5	2.2	71.2	0.2	0.4	0.2	0.3	0.3
Lungs	0.26	10.3	10.5	3.1	74.9	0.2	0.2	0.3	0.3	0.3
Heart	1.06	10.3	12.1	3.2	73.4	0.1	0.1	0.2	0.3	0.2
Intestine	1.03	10.6	11.5	2.2	75.1	0.1	0.1	0.1	0.2	0.1
Liver	1.06	10.2	13.9	3.0	71.6	0.2	0.3	0.3	0.2	0.3
Stomach	1.02	10.6	31.5	2.4	54.7	0.1	0.2	0.2	0.1	0.2
Kidneys	1.05	10.3	13.2	3.0	72.4	0.2	0.2	0.2	0.2	0.2
Spinal cord	0.98	11.5	64.4	0.7	23.1	0.1	0.1	0.1	—	—
Skin	1.09	10.0	20.4	4.2	64.5	0.2	0.1	0.2	0.3	0.1
Adipose tissue	1.05	11.4	59.8	0.7	27.8	0.1	—	0.1	0.1	—

The dimensions of the animal were derived on the basis of a study in which a mouse model was developed starting from the measurements of a real mouse about 25 g in weight.^27^ Then, a series of images of a female mouse taken with a laboratory‐based micro‐CT system were used to position the various organs inside the body in a realistic way.^28^

The mouse model described was implemented in the geometry of the shield set‐up (Figure 3b), presented in the previous section, and positioned inside the reactor thermal column. Monte Carlo simulations were conducted to calculate the dose‐rates in each animal's organs, and then possible parameters for future irradiations were determined (such as, irradiation time and reactor power which determine the neutron flux and fluence at the animal irradiation position). In particular, the contributions due to (i) the neutron capture reactions on 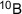 (α particle and 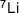 recoil nucleus, plus the 478 keV gamma ray produced in the 94% of reactions), assuming a concentration of 1 ppm in all tissues; (ii) the protons of the 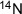
(n,p)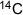 reactions; (iii) the recoil protons of the 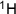
(n,n')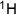 reactions; (iv) the 2.2 MeV of the 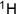
(${n,\gamma )}^{2}H$ reactions; and (v) to the background gamma rays in the thermal column, have been taken into account. In addition and for workers radiation protection evaluations, the presence of elements with a high probability of neutron activation was considered and thus an estimation of the expected neutron induced residual activities at the end of the irradiation was possible.

**FIGURE 4:**
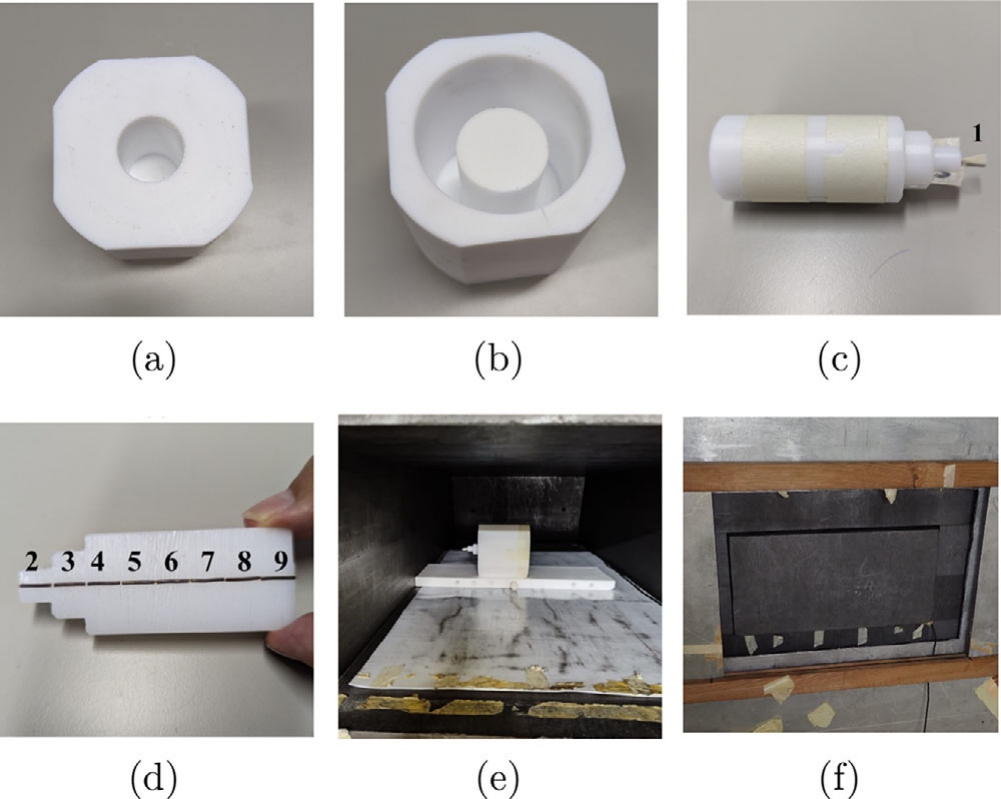
(a) Front view of the Teflon holder showing the central cavity designed to accommodate the polyethylene phantom; (b) backside of the same holder, where lithium carbonate powder can be loaded, thus surrounding the cavity visible in (a). (c) Polyethylene phantom used for irradiation with (d) the wire pieces inside. (e) Sample positioned in the thermal column of the Triga Mark II nuclear reactor (e) and the graphite block covering the entrance (f).

To carry out a preliminary experimental validation of the developed Monte Carlo model, a prototype for a single animal irradiation similar to those proposed in Figure 2a was designed and tested through neutron activation measurements. Specifically, a mouse phantom made of polyethylene, a tissue‐equivalent material, was constructed using a cylinder with a diameter of 3 cm and a length of 8 cm. Two smaller cylinders, 1 and 2 cm in diameter, respectively, were carved to reproduce the animal's head. The phantom was placed inside a Teflon holder in the shape of an octagonal prism (9 x 9 x 8.5 ${\mathrm{cm}}^{3}$) with four rounded edges; in one of the two bases, a cylinder 3 cm in diameter and 6 cm deep was drilled. On the opposite side, a cavity was created (volume of 207.32 ${\mathrm{cm}}^{3}$) to insert a 95% 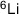
‐enriched lithium carbonate powder, a material used for neutron shielding (Figure 4a,b). 170 g of powder was used, and to increase its density, it was pressed by hand until it reached a value of approximately 0.82 g/${\mathrm{cm}}^{3}$.

The polyethylene mouse phantom was cut in half to create a groove along the central axis, in which a copper–gold wire was placed. The wire has a radius of 0.5 mm, a linear density of 71 mg/cm, and is composed of 98.45% copper and 1.55% gold in natural abundance. To monitor the neutron activation along the wire, it was cut into nine pieces of about 1 cm each before being placed in the groove (Figure 4c,d), with one piece placed outside the phantom (not shielded). Specifically, wires 1–3 represent the mouse head, 4–7 its central body, and 8–9 the caudal region.

The sample was placed inside the thermal column at a distance of 40 cm from the entrance (Figure 4e) with the protruding part of the phantom facing the left side of the column. To facilitate removal, the sample was placed on a 35 x 13 x 1.5 ${\mathrm{cm}}^{3}$ Teflon plate. With the aim of reducing the undue dose absorbed by the animal, before closing the irradiation chamber with the boral screen, a 40 x 20 x 10 ${\mathrm{cm}}^{3}$ block of graphite was placed at the entrance to reduce by 10% the 478 keV gamma flux (Figure 4f) which equals to approximately 10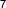 γ/s/${\mathrm{cm}}^{2}$ in an irradiation scenario with a reactor power of 250 kW and no graphite block.

The sample was irradiated for 30 min, after which it was removed from the cavity and the wires activities were measured with a High‐Purity Germanium detector (HPGe). To carry out the counting with the same cooling time and since the induced activities in the wires were very different depending on the position along the phantom, three measurement positions were chosen to optimize the counting procedure: The four innermost wires were placed in contact with the detector, the two middle ones at a distance of 6 cm and the three outermost at a distance of 10 cm. In all measurements the dead time remained below 5%.

To compute the saturation activity, it was necessary to determine the efficiency of the detector, therefore a 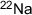 point source was used and the efficiency ε was calculated at the three counting positions. These values were weighted by a correction factor *h* since wires (i.e., an extended source) were in place during the measurements and therefore the self‐absorption of the emitted photons could not be negligible compared to the point‐like 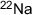 source. *h* is defined as the ratio between the efficiency that would have been obtained using a copper–gold wire ($\varepsilon_{wire}$) as calibration source and the efficiency calculated using the 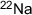 point source (ε); not having a reference source similar to wires in terms of size and composition, the simplest way was to calculate the *h* factor by means of Monte Carlo simulations with the MCNP6 code. For this purpose, two MCNP simulations were carried out with a very simple geometry consisting only of a cylinder reproducing the HPGe detector. In the first simulation the source was point‐like, while in the second the gamma rays were emitted inside a copper–gold wire with the same composition and dimensions used in the measurements. By calculating the current entering the cylinder base facing the source and performing the ratio between the two results, it was possible to determine the *h* factor in correspondence of the three distances from the detector and thus to better evaluate the saturation activity values.

In addition, after measuring the masses of each sample, the neutron fluxes at the nine wires were calculated.

The values of the saturation activities and fluxes obtained were compared with those calculated by means of Monte Carlo simulations. In particular, the entire sample was reproduced in the MCNP6 geometry and the reactions rates in correspondence of the wires were calculated. From these values, the neutron fluxes were determined, taking into account an additional correction factor due to the self‐absorption of neutrons. The latter was in fact calculated through a new simulation with the same experimental set‐up, but considering the wires filled with air, and computing the ratio between the reaction rate in air and the one in the gold–copper material.

## 3 RESULTS

### 3.1 Neutron and gamma flux distributions

In the designed shield set‐up and in the different irradiation positions within the thermal column of the Pavia Triga Mark II reactor, the distributions of the neutron and gamma fluxes in the three main compartments of the animal's body (head, middle, and caudal region) were evaluated in detail in air.

To present the data obtained as clearly and intuitively as possible, histograms were made of the total neutron fluxes in the head of the six mice as the position and shielding materials changed. In particular, it was observed that the minimum flux required for an NCT treatment was not reached at the head in pos. 1, therefore this irradiation position was no longer considered in the course of the study.

**FIGURE 5:**
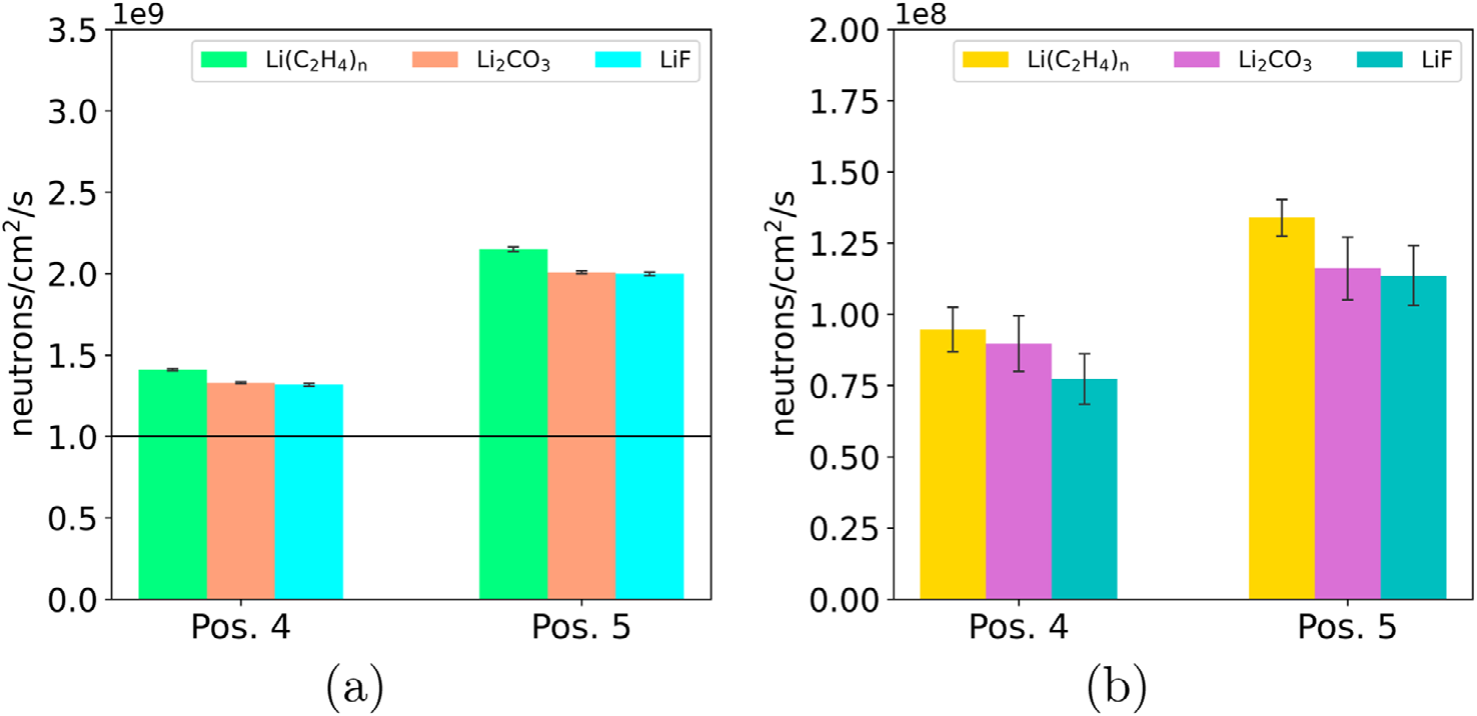
(a) Average neutron flux calculated in the cylinders representing the mouse head for each of the six holes of the shield, varying the materials and irradiation positions (pos. 4 and pos. 5). The black horizontal line in the graphs is inserted for the only purpose of aiding reading. It is placed at the threshold value (10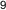 n/${\mathrm{cm}}^{2}$/s as prescribed by the NCT guidelines); (b) Average photon flux calculated in the nine cylinders representing the simplified mouse model and for each of the six holes present in the shielding, by varying the material and the irradiation position (pos. 4 and pos. 5).

The mean of the total neutron fluxes in the six mice head and related to different irradiation positions (Figure 2) are showed in Figure 5a. Additional data obtained varying the irradiation positions and considering different animals' compartments are presented in Supplementary Materials section, *Table* *S1*.

To better analyze the gamma flux behavior, the mean of total gamma fluxes in the animal's body were computed and showed in Figure 5b.

### 3.2 Experimental and computational neutron activation results

**FIGURE 6:**
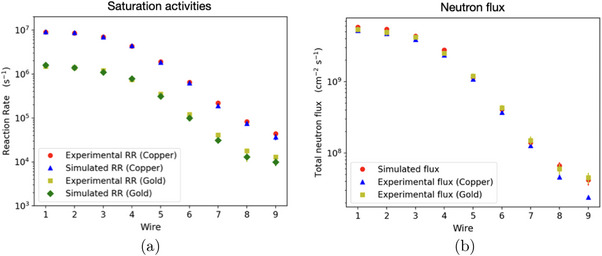
Computational and experimental (a) saturation activities and (b) neutron fluxes at the nine wires samples. The results are in a good agreement within 1σ, therefore allowing the computational set‐up model to be considered validated.

The mouse polyethylene phantom presented in Figure 4c was used to carry out Monte Carlo simulations and experimental measurement focused on neutron activation. The saturation activities (reaction rates) and neutron fluxes at the position of the nine wires were calculated (data reported in Supplementary Materials section, *Table* *S2*). In particular, Figure 6a,b aims to compare the results obtained experimentally and through MCNP6 Monte Carlo simulations.

### 3.3 Absorbed dose‐rates in the mice organs

**FIGURE 7:**
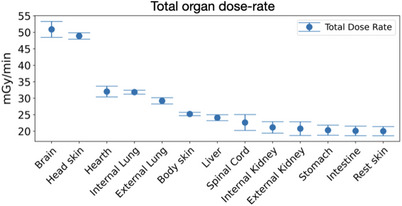
Mean values of total dose‐rates absorbed in each mice organs (from the outermost to the innermost) computed considering the designed neutron shield at irradiation position 4 (20 cm inside the cavity entrance). The errors bars represent the maximum and minimum values of the total dose rate found in the six mice.

**FIGURE 8:**
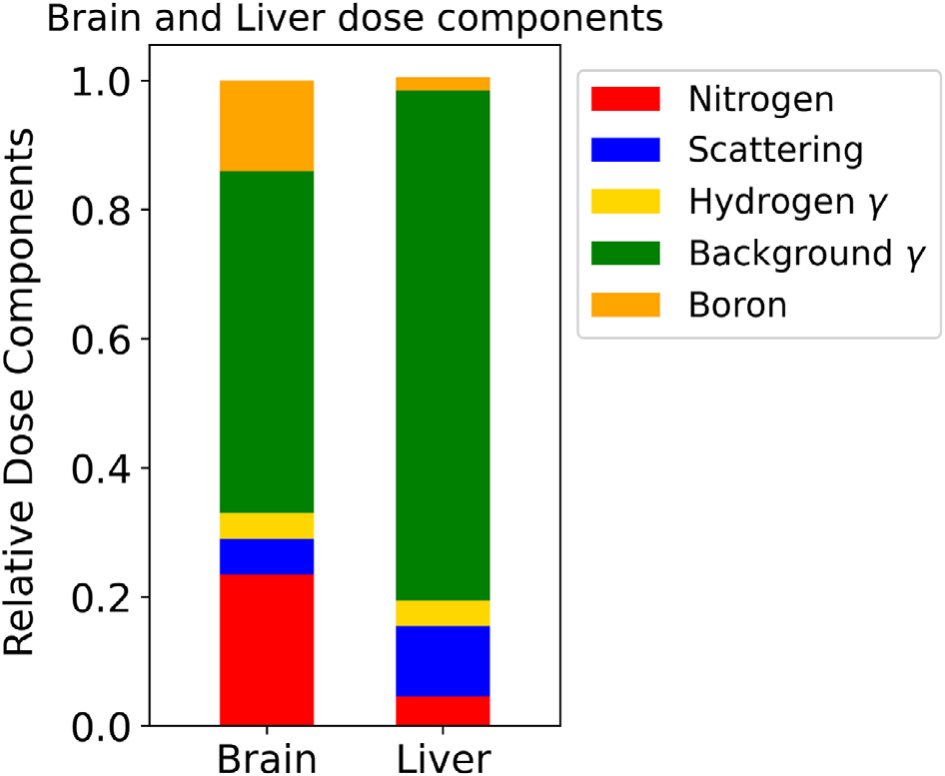
Percentage contribution of each dose component in brain and liver.

Using the model presented in Figure 3a, Monte Carlo simulations were conducted to estimate the dose‐rates under neutron irradiation by the field available in the modified thermal column of Pavia reactor. An isotropic distribution of 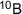 in the animal's body of 1 ppm was assumed, value chosen for practical calculation purposes, as it does not affect the simulations in terms of boron absorption effects. In addition, it was considered the Triga Mark II operating at its maximum power of 250 kW, with a neutron production rate of 1.9 x 10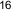 n/${\mathrm{cm}}^{2}$/s in the core region. The dose‐rates were calculated in each compartment of the mice; Figure 7 shows the total dose rates, averaged over the six animals, calculated in each organ while Figure 8 shows the percentage contribution of each dose component in the brain and liver, considered, respectively, as the target volume and the non‐target radiosensitive organ. Among the various compartments in particular, the liver was chosen because of its high metabolic (hepatic first‐pass effect) activity. Borated compounds, in fact, will be excreted through this organ leading to an increased 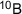 concentration in this region. Additional data are reported in Supplementary Materials section, *Table* *S3*.

### 3.4 Specific and saturation‐induced activity in animal tissues

Table 3 and Table 4 present, respectively, the results obtained from Monte Carlo simulations concerning the saturation activity and specific activity induced in animals following neutron irradiation. An irradiation time of 26 minutes and a waiting time of 15 min between the end of irradiation and the beginning of the counting measurement were assumed.

**TABLE 3:** Saturation activity in mice whole body computed through MCNP simulations.

Saturation Activity (Bq/g)							
	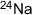	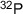	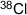	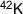	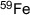	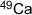	TOT
Mouse 1	1.502$\cdot 10^{5}$	3.448$\cdot 10^{4}$	2.622$\cdot 10^{4}$	1.190$\cdot 10^{4}$	12.67	3.330$\cdot 10^{-3}$	2.228$\cdot 10^{5}$
Mouse 2	1.508$\cdot 10^{5}$	3.463$\cdot 10^{4}$	2.639$\cdot 10^{4}$	1.197$\cdot 10^{4}$	13.05	3.339$\cdot 10^{-3}$	2.238$\cdot 10^{5}$
Mouse 3	1.482$\cdot 10^{5}$	3.379$\cdot 10^{4}$	2.592$\cdot 10^{4}$	1.171$\cdot 10^{4}$	12.65	3.347$\cdot 10^{-3}$	2.196$\cdot 10^{5}$
Mouse 4	1.509$\cdot 10^{5}$	3.451$\cdot 10^{4}$	2.646$\cdot 10^{4}$	1.202$\cdot 10^{4}$	13.55	3.335$\cdot 10^{-3}$	2.239$\cdot 10^{5}$
Mouse 5	1.490$\cdot 10^{5}$	3.407$\cdot 10^{4}$	2.594$\cdot 10^{4}$	1.170$\cdot 10^{4}$	11.23	3.349$\cdot 10^{-3}$	2.207$\cdot 10^{5}$
Mouse 6	1.488$\cdot 10^{5}$	3.392$\cdot 10^{4}$	2.605$\cdot 10^{4}$	1.176$\cdot 10^{4}$	12.82	3.339$\cdot 10^{-3}$	2.205$\cdot 10^{5}$
Mean	1.496$\cdot 10^{5}$	3.423$\cdot 10^{4}$	2.616$\cdot 10^{4}$	1.184$\cdot 10^{4}$	12.66	3.341$\cdot 10^{-3}$	2.219$\cdot 10^{5}$

**TABLE 4:** Specific activity in mice whole body computed through MCNP simulations.

Specific Activity [Bq/g]							
	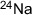	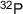	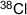	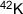	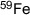	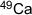	TOT
Mouse 1	2.944$\cdot 10^{3}$	30.21	7.614$\cdot 10^{3}$	2.818$\cdot 10^{2}$	3.563$\cdot 10^{-3}$	8.843$\cdot 10^{-4}$	1.087$\cdot 10^{4}$
Mouse 2	2.956$\cdot 10^{3}$	30.34	7.663$\cdot 10^{3}$	2.835$\cdot 10^{2}$	3.667$\cdot 10^{-3}$	8.849$\cdot 10^{-4}$	1.093$\cdot 10^{4}$
Mouse 3	2.905$\cdot 10^{3}$	29.60	7.526$\cdot 10^{3}$	2.773$\cdot 10^{2}$	3.556$\cdot 10^{-3}$	8.870$\cdot 10^{-4}$	1.074$\cdot 10^{4}$
Mouse 4	2.957$\cdot 10^{3}$	30.24	7.683$\cdot 10^{3}$	2.847$\cdot 10^{2}$	3.808$\cdot 10^{-3}$	8.839$\cdot 10^{-4}$	1.096$\cdot 10^{4}$
Mouse 5	2.921$\cdot 10^{3}$	29.86	7.531$\cdot 10^{3}$	2.770$\cdot 10^{2}$	3.158$\cdot 10^{-3}$	8.875$\cdot 10^{-4}$	1.076$\cdot 10^{4}$
Mouse 6	2.916$\cdot 10^{3}$	29.72	7.562$\cdot 10^{3}$	2.778$\cdot 10^{2}$	3.694$\cdot 10^{-3}$	8.850$\cdot 10^{-4}$	1.079$\cdot 10^{4}$
Mean	2.933$\cdot 10^{3}$	29.99	7.597$\cdot 10^{3}$	2.801$\cdot 10^{2}$	3.559$\cdot 10^{-3}$	8.854$\cdot 10^{-4}$	1.084$\cdot 10^{4}$

## 4 DISCUSSION

### 4.1 Flux evaluations related to irradiation set‐up

Considering the designed shielding set‐up (Figure 2a), a neutron flux greater than 10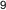 n/${\mathrm{cm}}^{2}$/s is achieved at the head (Figure 5a) with higher values in the case of the lithium polyethylene shield due to the neutron scattering on hydrogen atoms. At both irradiation positions 4 and 5, going deeper into the animals' compartments, it was recorded a decrease in flux by a factor of about 2.5 in the central region and by 50 in the caudal region, showing the effect of the modelled shielding. A flux of less than 10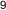 n/${\mathrm{cm}}^{2}$/s was recorded in correspondence of the first shielded cylinder which is protected taking into account the typical parameters working in an NCT treatment.

In the case of irradiation position 4 (Figure 5b) the gamma flux is 30% lower than position 5 due to its position within the thermal column, which is less affected by gamma radiation contamination. Position 4 is therefore the most suitable.

In terms of shielding materials, all materials have proven to perform well, but it must be considered that lithium polyethylene has a high hydrogen content (8.6%) so it is subject to radiative capture reactions resulting in the production of 2.2 MeV gamma which contributes to giving an undue dose to the animals. Both 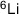
‐enriched lithium carbonate and lithium fluoride are generally supplied in powder form. However, lithium fluoride is also available as small crystalline pieces used in thermoluminescent detectors (TLDs), a format that is economically unfeasible and impractical when large quantities are needed. Given that both materials provide similar efficiency in thermal neutron attenuation, lithium carbonate was ultimately selected due to its lower affinity for moisture and its favorable sintering characteristics, which facilitate the fabrication of robust shielding components suitable for in vivo irradiation set‐ups.

### 4.2 MCNP model validation

Figure 6a shows the comparison between experimental measurements and Monte Carlo simulations concerning the saturation activities of copper–gold wires. The results are in agreement with each other; the values for wires 7, 8, and 9 deviate by an average of 13% in the case of copper and 25% in the case of gold but they are compatible within their errors.

The neutron fluxes at the wires determined experimentally from the reaction rates are in good agreement with those calculated from the simulations (Figure 6b). Experimental values for 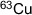 are slightly underestimated compared to those measured, in particular, by 10% from wires 1 to 7, 30% in wire 8 and 50% in wire 9. This is probably due to the approximations introduced during the flux calculation starting from the experimental reaction rate values, where macroscopic neutron capture cross sections were taken into account at an energy of 0.025 eV. Going deeper into the phantom, in fact, and considering the neutron shielding, the thermal component will be reduced and the epithermal one will prevail.

Nonetheless, these results allowed the irradiation set‐up model developed with the MCNP code to be considered validated.

### 4.3 Absorbed doses estimation in mice organs

Figure 8 shows the percentage contribution of each dose component to the total dose rate in the brain and the liver of the animal model assuming a 1 ppm concentration of 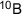 distributed uniformly. The liver was chosen as reference shielded organ to assess the efficiency of the designed shields. In particular, it was observed in both the organs the main contribution comes from the γ background (52.2% and 75%, respectively), the lowest is due to the 2.2 MeV gamma (4.5% and 6%). The contribution from neutron capture reactions on nitrogen is 23.5% and 6%, respectively, and elastic scattering is 5.5% and 11%. The fraction due to the neutron capture reactions on 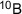 is not easily calculated due to the fact that the concentrations in each organ are not well known. For this reason, considering a uniform distribution of 1 ppm of 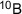, the contribution to the total dose rate is 14% in brain and 2% in liver, but it should be emphasized that this number may increase or decrease depending on the actual values of the 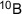 concentration.

Figure 7 shows the trend in total dose rates in each of the animal's organs as the depth in the animal's body increases. It can be seen that the highest value is recorded in the brain, as expected, and decreases with increasing depth; in particular, there is a reduction of about 40% in the lungs and heart and about 60% in the kidneys and stomach. These results demonstrate the high‐performance action of 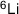
‐enriched lithium carbonate shielding designed, as also highlighted in previous studies.^19^

### 4.4 Neutron activation induced in animal tissues

*Tables* *3* and *4* show, respectively, the saturation and specific activities in each mouse calculated through Monte Carlo simulations. The highest contributions to the specific activity are due to 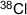 (7.597x10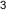 Bq/g) and 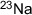 (2.933 x 10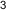 Bq/g) while the lowest is due to 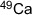 present only in the kidneys (8.854 x 10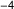).

### 4.5 in vivo irradiation protocol evaluation

The computational work presented in the previous sections enabled the development of an irradiation set‐up for future in vivo CENI experiments. Based on the obtained results and the estimated absorbed dose rates in the various animal organs, the maximum irradiation times were calculated with reference to the target organ, which was found to receive the highest dose, taking into account the reference absorbed dose. These evaluations were carried out in two different scenarios: (i) a possible future irradiation of wild‐type mice (not bearing senile plaques), designed to assess treatment‐induced toxicity, and (ii) a conventional BNCT context. The designed shielding, in fact, is also well suited for preclinical studies involving brain tumors; therefore, in this framework, the maximum irradiation time was estimated for the brain of healthy mice used as control groups, in order to ascertain the tolerance of a BNCT treatment, assuming 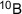
‐enriched BPA‐fructose (BPA‐F) adduct as the NCT agent.

In the first situation, the measured ppm concentrations in the brain and liver, the shielded organ assumed as reference, are very low, so the dose contribution due to background γ remains the predominant one. To establish possible irradiation times in relation to tolerable doses in the animal, previous studies^29^ conducted on mouse lung tumors were considered. Although the context and radiation field were different, the same shielding materials were tested and an absorbed dose of 2 Gy was observed in the control group. From this value and considering the reactor working at its maximum power of 250 kW, the irradiation times for the brain and liver were estimated to be 45 and 77 min, respectively.

It is important to emphasize that the extremely low brain concentration measured in wild‐type mice is not surprising given that the molecules developed in the NECTAR project maximize selective binding on β‐Amyloid aggregates and conversely should minimize internalization in brain cells that are not the target of treatment. The slightly higher uptake reported for the liver is due to its metabolic activity, in particular to the mechanisms activated to promote the excretion of the nanoparticles.

Considering the scenario of a possible brain tumor, RBE, CBE, and 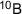 concentration values from the IAEA TecDoc^17^ and reference weighted dose from Kankaanranta *et al.'s* study^30^ were taken into account. The computed total weighted dose to the brain did not exceed 6 ${\mathrm{Gy}}_{w}$ in any mouse, with a mean value of approximately 3 ${\mathrm{Gy}}_{w}$. From these radiobiological data, an irradiation time of about 11 min was estimated to reach the upper limit accepted in^30^ in the healthy brain. Detailed data are provided in Supplementary Materials section, Tables 4S, 5S, and 6S.

## 5 CONCLUSION

The main goal of this study was to evaluate a dedicated and safe irradiation set‐up for small animals in the thermal column of the Triga Mark II nuclear reactor at the University of Pavia for future in vivo experiments in the NECTAR project scenario, evaluating a possible application in future research on brain tumor treatments.

After evaluating several irradiation positions and shielding materials, the configuration deemed most suitable involves placing the mice inside a shielding structure made of lithium carbonate enriched to 95% in 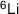, positioned 20 cm from the entrance of the thermal column. Monte Carlo simulations and preliminary activation measurements confirmed the effectiveness of this set‐up in minimizing gamma contamination and optimizing neutron exposure. In addition, a geometrical model of the mouse anatomy was developed and implemented in the MCNP6 code, enabling the estimation of organ‐specific absorbed doses. This allowed for the determination of safe irradiation times to be used in the protocol: 45 min for brain irradiations in the NECTAR scenario and 11 min for conventional BNCT brain tumor treatments, assuming a reactor power of 250 kW.

In conclusion, the developed irradiation protocol offers a concrete tool for a safe neutron doses delivery in small animal models, enabling the forthcoming in vivo experimental activity in the NECTAR project scenario. Beyond its application to Alzheimer's disease studies, this set‐up provides a solid basis for the preclinical evaluation of capture‐based therapies, potentially extending the conventional use of BNCT to a broader spectrum brain disorders.

## CONFLICT OF INTEREST STATEMENT

The authors declare no conflict of interest.

## Supporting information

Supporting Information
