# Supplementary material for: Capture‐enhanced neutron irradiation to treat Alzheimer's disease: Design of a small animal set‐up for future in‐vivo experiments
Source: Med Phys. 2025 Sep 1;52(9):e18062. doi: 10.1002/mp.18062 (PMC12402754; doi:10.1002/mp.18062)
Supplement: Supplementary file 1 — Supporting Information [file MP-52-0-s001.pdf]

Supplementary material section I

Table 1: *Values of the total neutron fluxes averaged over the six mice in the different body regions (head, central body and caudal region) as the shielding material changes.*

	Position 4			Position 5		
Material	Head $[n/cm^2/s]$	Central body $[n/cm^2/s]$	Caudal region $[n/cm^2/s]$	Head $[n/cm^2/s]$	Central body $[n/cm^2/s]$	Caudal region $[n/cm^2/s]$
Lithium Polythylene	$1.413 \cdot 10^9$ ± 0.5%	$0.235 \cdot 10^8$ ± 1%	$0.036 \cdot 10^8$ ± 3%	$2.150 \cdot 10^9$ ± 0.3%	$0.371 \cdot 10^8$ ± 0.5%	$0.0590 \cdot 10^8$ ± 2%
Lithium Carbonate	$1.329 \cdot 10^9$ ± 0.5%	$0.203 \cdot 10^8$ ± 1%	$0.031 \cdot 10^8$ ± 3%	$2.008 \cdot 10^9$ ± 0.3%	$0.324 \cdot 10^8$ ± 1%	$0.0535 \cdot 10^8$ ± 2%
Lithium Fluoride	$1.317 \cdot 10^9$ ± 0.5%	$0.197 \cdot 10^8$ ± 1%	$0.0274 \cdot 10^8$ ± 3%	$1.998 \cdot 10^9$ ± 0.3%	$0.314 \cdot 10^8$ ± 1%	$0.0464 \cdot 10^8$ ± 2%

Table 2: *Results of experimental measurements and MCNP simulations of reaction rates.*

Wire	Experimental RR (Copper) $[s^{-1}]$	Simulated RR (Copper) $[s^{-1}]$	Experimental RR (Gold) $[s^{-1}]$	Simulated RR (Gold) $[s^{-1}]$
1	$(9.0 \pm 0.2) \cdot 10^6$	$(9.1 \pm 0.1) \cdot 10^6$	$(1.5 \pm 0.2) \cdot 10^6$	$(1.60 \pm 0.04) \cdot 10^6$
2	$(8.5 \pm 0.2) \cdot 10^6$	$(8.7 \pm 0.1) \cdot 10^6$	$(1.4 \pm 0.2) \cdot 10^6$	$(1.40 \pm 0.04) \cdot 10^6$
3	$(7.0 \pm 0.2) \cdot 10^6$	$(7.0 \pm 0.1) \cdot 10^6$	$(1.2 \pm 0.2) \cdot 10^6$	$(1.10 \pm 0.04) \cdot 10^6$
4	$(4.3 \pm 0.1) \cdot 10^6$	$(4.43 \pm 0.1) \cdot 10^6$	$(7.40 \pm 0.05) \cdot 10^5$	$(7.80 \pm 0.06) \cdot 10^5$
5	$(1.91 \pm 0.06) \cdot 10^6$	$(1.85 \pm 0.05) \cdot 10^6$	$(3.5 \pm 0.03) \cdot 10^5$	$(3.10 \pm 0.03) \cdot 10^5$
6	$(6.50 \pm 0.04) \cdot 10^5$	$(6.20 \pm 0.03) \cdot 10^5$	$(1.20 \pm 0.01) \cdot 10^5$	$(1.0 \pm 0.01) \cdot 10^5$
7	$(2.20 \pm 0.01) \cdot 10^5$	$(1.90 \pm 0.02) \cdot 10^5$	$(4.1 \pm 0.005) \cdot 10^4$	$(3.1 \pm 0.005) \cdot 10^4$
8	$(8.3 \pm 0.005) \cdot 10^4$	$(7.5 \pm 0.009) \cdot 10^4$	$(1.8 \pm 0.003) \cdot 10^4$	$(1.3 \pm 0.002) \cdot 10^4$
9	$(4.4 \pm 0.002) \cdot 10^4$	$(3.7 \pm 0.006) \cdot 10^4$	$(1.3 \pm 0.002) \cdot 10^4$	$(1.0 \pm 0.002) \cdot 10^4$

Table 3: *Aborbed dose-rates mean values computed in each anatomical compartment considering the neutron shield at the irradiation position 4.*

	^{14}N(n,p)^{14}C [mGy/min]	^{1}H(n,n')^{1}H [mGy/min]	^{1}H(n,γ)^{2}H [mGy/min]	γ background	^{10}B(n,α)^{7}Li [mGy/min]
Brain	11.6 ± 0.2	2.8 ± 0.7	2.17 ± 0.1	25.9 ± 1.4	6.9 ± 0.1
Head Skin	13.2 ± 0.2	3.89 ± 0.8	2.88 ± 0.1	21.1 ± 0.3	7.86 ± 0.1
Central Body Skin	1.13 ± 0.03	2.80 ± 0.43	4.37 ± 0.34	16.30 ± 0.45	0.67 ± 0.02
End of Body Skin	11.6 ± 0.2	0.013 ± 0.003	3.78 ± 0.58	16.0 ± 0.04	0.28 ± 1.21
Internal Lung	4.51 ± 0.10	3.1 ± 0.9	0.32 ± 0.03	21.8 ± 0.3	0.97 ± 0.59
External Lung	3.92 ± 0.16	2.3 ± 1.1	0.29 ± 0.05	20.9 ± 0.8	1.70 ± 0.12
Heart	5.13 ± 0.14	1.9 ± 0.7	0.55 ± 0.03	22.3 ± 1.1	2.08 ± 0.06
Liver	1.03 ± 0.07	2.4 ± 0.7	1.03 ± 0.03	18.8 ± 0.7	0.45 ± 0.03
Spinal Cord	0.43 ± 0,04	2.8 ± 0.7	0.06 ± 0.01	18.4 ± 1.8	0.81 ± 0.08
Stomach	0.27 ± 0.03	2.6 ± 2.0	0.14 ± 0.03	17.1 ± 1.6	0.14 ± 0.02
Internal Kidney	0.30 ± 0.07	3.2 ± 2.5	0.13 ± 0.02	17.3 ± 1.3	0.14 ± 0.03
External Kidney	0.29 ± 0.08	3.1 ± 1.8	0.12 ± 0.02	17.2 ± 0.7	0.12 ± 0.03
Intestine	0.13 ± 0.03	3.0 ± 1.0	0.64 ± 0.04	16.2 ± 0.7	0.08 ± 0.01

Supplementary material section II

Table 4: *Radiobiological parameters and estimated absorbed dose for the NECTAR irradiation scenario.*

Organ	ppm	T_{irr} (min)	Absorbed Dose (Gy)	Reference Absorbed Dose (Gy)
Brain	0.09	45	1.94 (1.85-1.97)	2
Liver	4	77	1.93 (1.84-1.99)	2

Table 5: *Radiobiological parameters for the brain tumour BNCT scenario.*

ppm	RBE_p	CBE
16	2.9	3.8

Table 6: *Biological weighted doses estimated for the brain tumor BNCT scenario.*

Organ	ppm	T_{irr} (min)	Weighted Dose (Gy_w)	Reference Weighted Dose (Gy_w)
Brain	16	11	5.38 (5.34-5.56)	6
Liver	4	11	0.40 (0.37-0.44)	8.6
